# UV radiation affects antipredatory defense traits in *Daphnia pulex*

**DOI:** 10.1002/ece3.6999

**Published:** 2020-11-21

**Authors:** Franceen Eshun‐Wilson, Raoul Wolf, Tom Andersen, Dag O. Hessen, Erik Sperfeld

**Affiliations:** ^1^ Department of Bioscience University of Oslo Oslo Norway; ^2^ Norwegian Institute for Water Research (NIVA) Oslo Norway; ^3^ Animal Ecology Zoological Institute and Museum University of Greifswald Greifswald Germany

**Keywords:** aquatic ecosystem, Bayesian statistics, *Chaoborus*, Kairomone, MCMC chains, phenotypic plasticity, predator–prey interaction, UV‐A, zooplankton

## Abstract

In aquatic environments, prey perceive predator threats by chemical cues called kairomones, which can induce changes in their morphology, life histories, and behavior. Predator‐induced defenses have allowed for prey, such as *Daphnia pulex*, to avert capture by common invertebrate predators, such as *Chaoborus* sp. larvae. However, the influence of additional stressors, such as ultraviolet radiation (UVR), on the *Daphnia–Chaoborus* interaction is not settled as UVR may for instance deactivate the kairomone. In laboratory experiments, we investigated the combined effect of kairomones and UVR at ecologically relevant levels on induced morphological defenses of two *D. pulex* clones. We found that kairomones were not deactivated by UVR exposure. Instead, UVR exposure suppressed induced morphological defense traits of *D. pulex* juveniles under predation threat by generally decreasing the number of neckteeth and especially by decreasing the size of the pedestal beneath the neckteeth. UVR exposure also decreased the body length, body width, and tail spine length of juveniles, likely additionally increasing the vulnerability to *Chaoborus* predation. Our results suggest potential detrimental effects on fitness and survival of *D. pulex* subject to UVR stress, with consequences on community composition and food web structure in clear and shallow water bodies.

## Graphical abstract

In aquatic environments, prey perceive predator threats by chemical cues called kairomones, which can induce changes in their morphology, life histories, and behavior. Predator‐induced defenses have allowed for prey, such as *Daphnia pulex*, to avert capture by common invertebrate predators, such as *Chaoborus* sp. larvae. The influence of additional stressors, such as ultraviolet radiation (UVR), on the Daphnia–Chaoborus interaction is not settled as UVR may deactivate the kairomone. In laboratory experiments, we investigated the combined effect of kairomones and UVR at ecologically relevant levels on induced morphological defenses of two *D. pulex* clones. We found that kairomones were not deactivated by UVR exposure. Instead, UVR exposure suppressed induced morphological defense traits of *D. pulex* juveniles under predation threat by generally decreasing the number of neckteeth and especially by decreasing the size of the pedestal beneath the neckteeth.

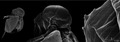

## 1 INTRODUCTION

Studies on predator–prey dynamics have provided powerful insights into the ability of prey organisms to respond and adapt to predators by morphological, behavioral, or life‐history responses (Dodson, 1989; Ghalambor et al., 2015; Hammill et al., 2008). The outcome of these responses may again affect population dynamics of both predators and prey (Abrams, 1986; Marrow et al., 1992). The coevolution of predator–prey interactions is fueled by the antagonistic biotic interaction, favoring the multifaceted response of defense traits (Clay & Kover, 1996; Marrow et al., 1992). The development of defense traits may be costly, making it beneficial for prey to develop defenses during periods of predation threat, especially if the predator occurs only seasonally or unpredictably (Tollrian & Harvell, 1999). Predator‐induced defenses are prevalent among many taxa (Lass & Spaak, 2003; Tollrian & Harvell, 1999), allowing for prey organisms to respond to varying risk of predation (Christjani et al., 2016; Dennis et al., 2011).

**FIGURE 1:**
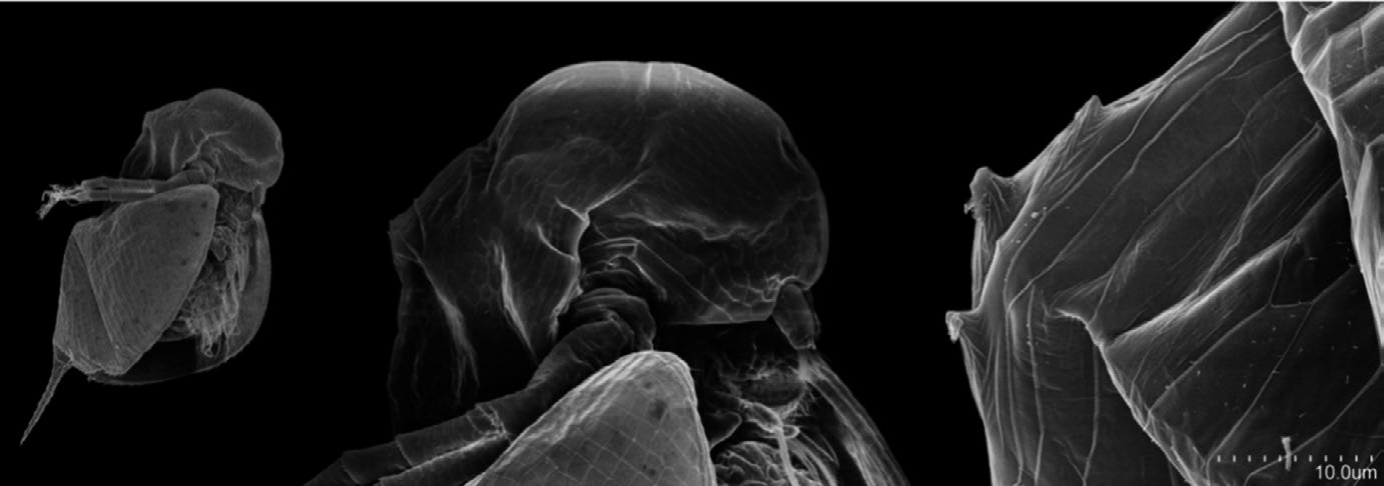
Scanning electron microscopy (SEM) images showing a 2nd instar juvenile male of the *Daphnia pulex* clone P5 that has been exposed to kairomones of *Chaoborus flavicans* larvae. Image includes full body, headshot, and close‐up of three induced neckteeth with pedestal of score type B. Photo‐credit: Jannicke Wiik‐Nielsen, Norwegian Veterinary Institute

*Daphnia pulex* (Leydig 1860) has been widely used as a model organism to explore shifts in life‐history, morphological, and behavioral‐defensive traits in response to predatory info‐chemical cues, named kairomones (e.g., Boeing et al., 2006; Krueger & Dodson, 1981; Tollrian, 1995). *Chaoborus* sp. larvae, also known as the phantom midge larvae or glass worm, are common predators of *Daphnia* species. They prey mostly on juveniles of *D. pulex* and other smaller *Daphnia* species due to gape limitation (Sell, 2000; Tollrian, 1995). One striking feature of a morphological defense in juvenile *D. pulex* is the development of neckteeth (neck spines) and a neck‐pedestal beneath the neckteeth (Figure 1) that are induced by kairomones released from actively feeding *Chaoborus* larvae and that serve to protect against predation (Krueger & Dodson, 1981; Riessen & Trevett‐Smith, 2009; Tollrian & Dodson, 1999). *D. pulex* can possess interclonal differences in response rates of neckteeth and pedestal induction based on kairomone concentrations (Carter et al., 2017; Christjani et al., 2016; Dennis et al., 2011; Hammill et al., 2008). Other induced morphological defenses in *D. pulex* include increases in body size, elongation of the tail spine, and strengthening of the carapace (Riessen et al., 2012). Increases in body size are linked to greater swimming speed and thus a higher escape and avoidance rate (Tollrian, 1993).

Defensive traits are costly and may thus be involved in trade‐off strategies to ensure energy allocations to growth, reproduction, and survival (Hammill et al., 2008). The dynamic nature of the induction of neckteeth in *Daphnia* is the result of a form of optimization between predation risk and protective investments with benefits and costs of this trait likely limited to juvenile stages due to the gape limitation of the predator *Chaoborus* (Hammill et al., 2008; Tollrian, 1995). Recorded costs of neckteeth induction include longer development time for offspring, reduced survival, and reduced reproductive success (Hammill et al., 2008; Riessen, 2012; Tollrian, 1995). Such costs could lead to greater vulnerability to other environmental stressors and thus impose trade‐offs.

Recent studies have shown that shifts in environmental conditions can negatively impact *D. pulex's* ability to induce neckteeth in the presence of predatory cues (Rautio & Tartarotti, 2010; Riessen et al., 2012; Weiss, Pötter, et al., 2018). Changes in water chemistry, such as low calcium concentrations, resulted in decreased neckteeth formation, decreased body size, and weakening of the carapace in *D. pulex* (Riessen et al., 2012). Similarly, *p*CO_2_‐dependent freshwater acidification impaired predator cue perception and reduced the ability of *D. pulex* to form neckteeth (Weiss, Pötter, et al., 2018). Solar ultraviolet radiation (UVR) is another environmental stressor that may increase vulnerability to predation by decreasing induction of antipredatory defenses (Sterr & Sommaruga, 2008).

UVR is an ever‐present stressor in clear or shallow aquatic habitats, yet changing in intensity seasonally. This is leading to varying synthesis or accumulation of photoprotective compounds (e.g., melanin, carotenoids, and other pigments) in planktonic crustaceans (e.g., Herbert & Emery, 1990; Hylander et al., 2015). Stress imposed by UVR could lead to life strategy trade‐offs. The resistance of the melanic *D. dadayana* to UVR was however not affected by the investment in antipredatory defenses, implying no trade‐off between predation threat and UVR (Wolinski et al., 2020). The allocation of energy to defenses against UVR exposure may impact the prey's ability to defend against other environmental stressors, such as low calcium availability, predation, or infection (Hessen & Rukke, 2000; Hoverman & Relyea, 2009). Laboratory and field studies have shown organisms to be negatively affected when exposed to high intensities of UVR (Hansson et al., 2007; Hessen et al., 1995; Kim et al., 2009). UVR has been linked to DNA damage, reduced growth rates, and decreased fecundity (Rautio & Tartarotti, 2010). It may also cause detrimental effects by producing intracellular or ambient reactive oxygen species (ROS) formation in natural systems (Cullen & Neale, 1994; Wolf et al., 2017).

An alternative mechanism to suppress inducible defenses of prey may be the photochemical degradation of kairomones by UVR. Sterr and Sommaruga (2008) have shown that strong UVR exposure can degrade the *Chaoborus* kairomones, rendering the chemical cue ineffective in inducing defensive traits in *D. pulex*. The relevance of this mechanism in natural systems is unclear as kairomones are constantly produced when the predator is present. Also, high UVR exposure may not coincide with the presence of the predator in the system. However, it remains to be tested how UVR exposure of the animals affects their capability to develop defense traits induced by *Chaoborus* kairomones.

We here assess the effects of ecologically relevant UVR levels on defensive traits of two *D. pulex* clones from distinct geographical locations without and with exposure to kairomones in a two‐by‐two factorial design. We hypothesized that UVR will limit induced morphological defenses in *D. pulex* juveniles by either degradation or denaturation of the kairomone or due to a direct UVR‐related stress response. We also hypothesized interclonal differences due to clones originating from ponds with greatly differing light exposure.

## 2 MATERIALS AND METHODS

### 2.1 Clone collection

Two *D. pulex* clones from distinct geographical locations were collected in 2017 in southern Norway. The first clone, named “UNI”, was collected on September 22nd, 2017, from an artificial reservoir pond next to the Biology department building of the University of Oslo (GPS: 59.937767, 10.722368). This pond (~10 × 10 m, max. depth ~2 m) is heavily shaded by beech trees during the growing season and usually drained during the winter season. The water color during sampling was lightly brown due to litter input from beech leaves. Plankton net hauls during sampling showed no occurrence of *Chaoborus* larvae, but in certain years the pond may hold high densities of *Chaoborus*. The second clone, named “P5”, was collected on September 16th, 2017, from a very small rock pool (~1 × 2 m, max. depth ~ 0.3 m) on an island in south‐eastern Norway (GPS: 59.098405, 11.198153). The rock pool often dries out and reoccurs during summer in response to rainfall regimes. It has a distinct brownish water color due to humic substance input from surrounding vegetation. This pond has been sampled during different seasons and years, and *Chaoborus* larvae have never been observed. The two clones (P5 and UNI) were kept for several generations in stock cultures without being exposed to *Chaoborus* kairomones or UVR before being used in experiments from July 1st, 2018, to February 2nd, 2019.

### 2.2 General culture conditions

Both *D. pulex* clones were kept at 21 ± 1°C in a temperature‐controlled climate room with a 16:8 hr light:dark cycle. The daphnids were cultured in ADaM medium (Klüttgen et al., 1994), modified by using 0.05 times the recommended SeO_2_ concentration, and fed daily with 2 mg C L^−1^ of the green algae *Chlamydomonas reinhardtii* (CC‐1690 wild‐type mt+, Chlamydomonas Resource Center). *C. reinhardtii* were maintained in oxygenated semicontinuous cultures on modified WC (Wright Cryptophyte) medium with vitamins (Guillard, 1975) at 21 ± 1°C with constant photosynthetically active radiation (PAR) lighting and harvested during the exponential phase of algae growth (replaced every 14 days). Algae were centrifuged at 1,740 *g* for 10 min (Eppendorf Centrifuge 5810 R), and the WC medium was replaced with fresh ADaM medium before feeding to *D. pulex* cultures. During the experiment, daphnids were fed lower amounts of food (0.5 mg C L^−1^) once a day to limit the growth of bacteria that may degrade kairomones (Tollrian, 1993). Carbon concentrations of algal suspensions were estimated from photometric light extinction (800 nm, Shimadzu UV 160‐A, Japan) using a previously determined carbon‐light extinction conversion equation.

### 2.3 Preparation of kairomone extract

The kairomone was extracted from frozen *Chaoborus flavicans* larvae (Akvarie Teknik, Sweden) following a protocol adapted from Hebert and Grewe (1985) to produce a large amount of kairomone with identical activity. 100 g frozen *Chaoborus* larvae were boiled in 200 ml water for 10 min and larvae were removed afterward using a mesh gauze. Particles were removed by centrifugation (3,100 *g*, 20 min) and subsequent filtration (0.1 µm, Vacuum filtration, Filtropur V50 500 ml, Sarstedt). The extract was aliquoted in 1.5 ml tubes and stored at −20°C until use in experiments. In other studies, such extracts have been further purified by solid‐phase extraction (Tollrian, 1995; Tollrian & von Elert, 1994), but this was not necessary in our case as we did not observe adverse effects of the unpurified extract on our *D. pulex* clones in previous experiments, and the extract was highly effective in inducing neckteeth (see below).

### 2.4 UVR setup

In the experiments, animals were exposed to two groups of light treatment: a photosynthetically active radiation (PAR) treatment as a control, and a UVR treatment. For the PAR treatment, two 36‐W fluorescent lamps were installed 15 cm above the open glass jars containing *D. pulex* and set to a 16:8 hr light:dark cycle to mimic natural light conditions (400–700 nm). The UVR treatment followed the experimental setup described by Wolf and Heuschele (2018). UV‐A radiation lamps (UVA‐340, Q‐Lab, Westlake, USA) were selected due to close simulation of sunlight in the wavelength region from 300 to 365 nm with peak emission at 340 nm (total range: 295–400 nm) (Q‐Lab 2019, www.q‐lab.com). The surface area of jars in both treatment groups was exposed to the same total light intensity (1,900 lux). Light intensity of PAR treatment and UV‐A radiation lamps was measured using a spectroradiometer (SpectraPen LM‐500‐UVIS, Photon Systems, Instruments, Drásov, Czech Republic). Further information and details on the UVR setup, for example, the spectral distribution of the photon flux of the used lamps, are given in Wolf and Heuschele (2018).

### 2.5 Experiment testing the effect of UVR on kairomone effectivity

We first conducted an experiment to (a) demonstrate that our kairomone extract effectively induced neckteeth formation as well as to (b) investigate the hypothesis that UVR may limit neckteeth induction by UVR making the kairomone ineffective. A study by Sterr and Sommaruga (2008) showed that UVR exposure of *Chaoborus* kairomones for 5 to 10 hr reduced neckteeth induction. In our study, the integrity of kairomone suspensions was tested for different time intervals with the following three treatments (with 8 replicates/jars per treatment): ADaM medium with kairomones exposed to UVR (340–400 nm), medium with kairomones exposed to PAR (400–700 nm), and a control with medium that contained no kairomones and was kept in the dark. Kairomone solutions were prepared by adding 60 µl of the kairomone extract into 50 ml glass jars containing 40 ml ADaM medium and 0.5 mg C L^−1^ *C. reinhardtii*. The amount of kairomone added would correspond to ~75 *Chaoborus* larvae L^−1^, using a conversion factor between the kairomone extract and *Chaoborus* density established by Hammill et al. (2008). The jars containing the kairomone solutions and the control were subjected to the respective treatments for 2, 4, 6, and 8 hr with no daphnids present. Juvenile *D. pulex* induce neckteeth when the *Chaoborus* kairomone is present during the late phase of the embryonal development in the brood pouch of the mothers (Krueger & Dodson, 1981; Naraki et al., 2013; Weiss et al., 2016). After each time period, females of the UNI clone with developing offspring in their brood pouch were placed individually in jars of the different treatment groups (UVR, PAR, and control treatments, respectively), with 2 individuals for each group and time interval. Five to ten released offspring juveniles per mother were inspected under the microscope at their 2nd instar to count the number of neckteeth and score the pedestal (see below).

### 2.6 Experiment testing the direct effect of UVR on *Daphnia*

In a second, larger experiment we tested whether exposure of egg‐bearing mothers and offspring to UVR would affect kairomone‐induced neckteeth formation in the juveniles. Both *D. pulex* clones were exposed to the following four treatments in a full factorial design: without UVR or kairomone exposure (control), kairomone exposure without UVR exposure, UVR exposure without kairomone exposure, and UVR and kairomone exposure. *D. pulex* females of both clones carrying the 4th clutch in their brood pouch were used for the experiment (5–7 mothers per treatment). These mother individuals were placed individually in transparent 50 ml open glass jars filled with 40 ml ADaM medium and 0.5 mg C L^−1^ *C. reinhardtii* and were exposed to UVR and kairomones depending on treatment. Kairomone treatments were prepared by adding 60 µl of the kairomone extract to the jars. UVR and non‐UVR treatment groups were exposed to UVR and PAR light, respectively, in 16:8 hr light:dark cycles. Mother individuals of all treatment groups were transferred daily to freshly prepared kairomone and food suspensions until release of their 4th clutch juveniles. The mothers were removed, and juvenile clutch groups were kept in the same treatments until reaching the 2nd instar in order to mimic natural conditions of UVR and kairomone exposure. Juveniles were inspected daily alive using a microscope for counting neckteeth, scoring pedestals, and taking photographs of the full body using a computer‐aided camera for later length measurements (see below).

### 2.7 Scoring of morphological defense traits and length measurements

**FIGURE A1:**
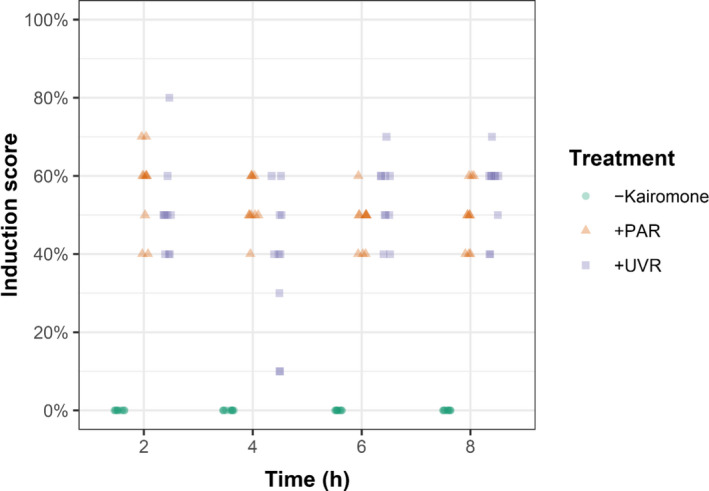
Neckteeth induction score (%) in 2nd instar offspring of *Daphnia pulex* mothers of clone UNI. In the UVR and PAR treatment, media‐filled jars with added kairomone extract have been exposed to UVR and PAR, respectively, for 2, 4, 6, and 8 hours before placing *D. pulex* mothers in the jars. In the control treatment ‘–Kairomone’, *D. pulex* mothers were kept in jars in the dark without addition of kairomone extract. Data of individual offspring juveniles (*n* = 10 per treatment group and time interval) are shown. A: no pedestal, B: small pedestal, C: large pedestal

Neckteeth, that is, small spines at the dorsal head margin, were counted on live individuals of *D. pulex* juveniles in the 1st and 2nd instar using a microscope (Nikon Eclipse E200) with 100× magnification. At the base of the neckteeth, a pedestal of varying size can develop and was scored in a categorial way with “A” when absent, “B” when small, and “C” when large. Individuals were then photographed at 40× magnification for later length measurements (see below) with a microscope‐mounted Nikon camera (DS‐5M). From the neckteeth counts and pedestal score, a neckteeth induction score has been calculated according to Tollrian (1993). However, we focus our analyses on the neckteeth count and pedestal score separately, because both defense traits varied somewhat in their treatment responses. The results of the neckteeth induction scores are presented in the Appendix (Figures A1, A3).

Body length, body width, and tail spine length of *D. pulex* juveniles were measured from the photographs using ImageJ and a landmark approach (Sperfeld et al., 2020). Body length was calculated as the distance between the top of the head and the base of tail spine, body width between the ventral midpoint and dorsal midpoint, and tail spine length between the base and the tip of the tail spine (see Sperfeld et al., 2020 for further details). Technical difficulties caused by image file corruption limited the number of measurements in some treatment groups of instar 2 juveniles.

### 2.8 Statistical analyses

Our data are naturally organized into a grouping structure due to using multiple neonates from the same mother. We accounted for this maternal dependency by using hierarchical models (also called multi‐level models, or mixed effects models) for all statistical analyses. Since mothers were numbered consecutively within each clone and treatment combination, we constructed unique mother identifiers as clone by treatment by mother ID interactions.

We used a Bayesian approach for all model fitting, using the *brms* package (Bürkner, 2017). This package uses standard R formula syntax to specify the model, which is then translated into code that can be run by the *Stan* environment for Bayesian computing (Carpenter et al., 2017). *Stan* is a powerful computing platform that uses a modified Hamiltonian Monte Carlo algorithm to sample efficiently from the posterior distribution of a Bayesian model. All statistical analyses were conducted using the open‐source statistical software R statistics (R Core Team, 2017).

To investigate whether UVR and PAR exposure affected the effectivity of the kairomone extract to induce neckteeth and pedestals, a bivariate Bayesian regression model was fit to the data of the “experiment testing the effect of UVR on kairomone effectivity.” The data of all time points were pooled, pedestal scores were assumed to come from a cumulative distribution with two thresholds, and neckteeth count was supposed to come from a binomial distribution with five trials. The effect of each treatment (control, PAR, and UVR) was implemented as linear predictor. Standard normal distribution priors with an average of zero and a standard deviation of ten were chosen. Five parallel Monte Carlo Markov chains were run for 4,000 iterations each, whereof half was used for warmup, resulting in 10,000 posterior samples. Model parameters were further investigated in Bayesian hypothesis testing to quantify potential differences in kairomone effectivity between treatments (results of this are given in Appendix: Table A1). This was based on evidence ratios, that is, the Bayes factor between the hypothesis (*H*_0_) and its alternative (*H*_1_), computed via the Savage–Dickey density ratio method (Dickey, 1971; Verdinelli & Wasserman, 1995). This was done using the *hypothesis()* function of the *brms* package.

The *brms* package (and implicitly *Stan*) has the functionality for representing multivariate models, that is, models with more than one dependent variable. We use this feature in two different ways for the morphometric and inducible defense trait data sets. The morphometric data (body length and width, and tail spine length) contain an inherent correlation structure. Instead of fitting separate models for each morphometric trait, we fit one multivariate model for all three traits under the assumption that they can be described by a (zero truncated) multivariate Gaussian distribution. A full model representing all possible interactions (Clone, Instar, Kairomone, UVR) was fit to the entire data. After initial models with this quadruple interaction (Clone:Instar:Kairomone:UVR) were unidentifiable (*R*_hat_ value > 1.05, low effective sample sizes), which is a strong indicator for overfitting, this quadruple interaction was removed from the model.

The two aspects of neckteeth induction, pedestal score and neckteeth count, are usually combined into a single 0%–100% index (Tollrian, 1993). Since this index has challenging statistical properties (constrained to the 0%–100% interval, often with an overabundance, i.e., inflation of zeros), we chose instead to represent the pedestal scores and neckteeth count as separate components of a bivariate model. We represented the pedestal score as a factor variable with ordered levels, that is, an ordinal variable based on Tollrian’s (1993) recommendations, ranging from A (no pedestal) to C (large pedestal), while the neckteeth count was treated as count data with possible values from 0 to 5, that is, a quintuple binomial trial. We modeled both the ordinal pedestal score probabilities and the neckteeth count probabilities with logistic links, such that predicted zeros mean 50% probability, while prediction approaching ± infinity means very high/low probability. The bivariate models including neckteeth count and pedestal score were fitted separately for each instar, as neckteeth have been observed to occur in the 1st instar even without kairomone exposure (e.g., Naraki et al., 2013; Weiss et al., 2016; and also in this study), suggesting canalization rather than induction (Waddington, 1942; Weiss et al., 2016), and thus might represent different causal links in each instar. For both instars, full models were fit (Clone, Kairomone, UVR); again, inclusion of the triple interaction (Clone:Kairomone:UVR) made the models unidentifiable, and the triple interaction was removed for the final models.

Bayesian models consist of two parts: a likelihood of observing the data given the parameter values and a prior probability distribution for these parameters. We experienced convergence problems for all models when using so‐called uninformative priors, which are the default for *brms*. With weakly informative priors, that is, Gaussian distributions with mean zero and standard deviation equal to 10 for all model coefficients, we experienced no convergence problems after removing maximum interaction terms (see above), and all MCMC chains converged with *R*_hat_ < 1.01 (Vehtari et al., 2019). We used 4 MCMC chains, each with 2,000 iterations and 1,000 warmups, totaling 4,000 postwarmup samples of the posterior distributions. These samples are then used to compute credible intervals for model parameters. Detailed information on data organization, model, and prior specification, etc. can be found in the publicly available scripts (see Data Availability Statement).

## 3 RESULTS

### 3.1 Kairomone effectivity

**FIGURE 2:**
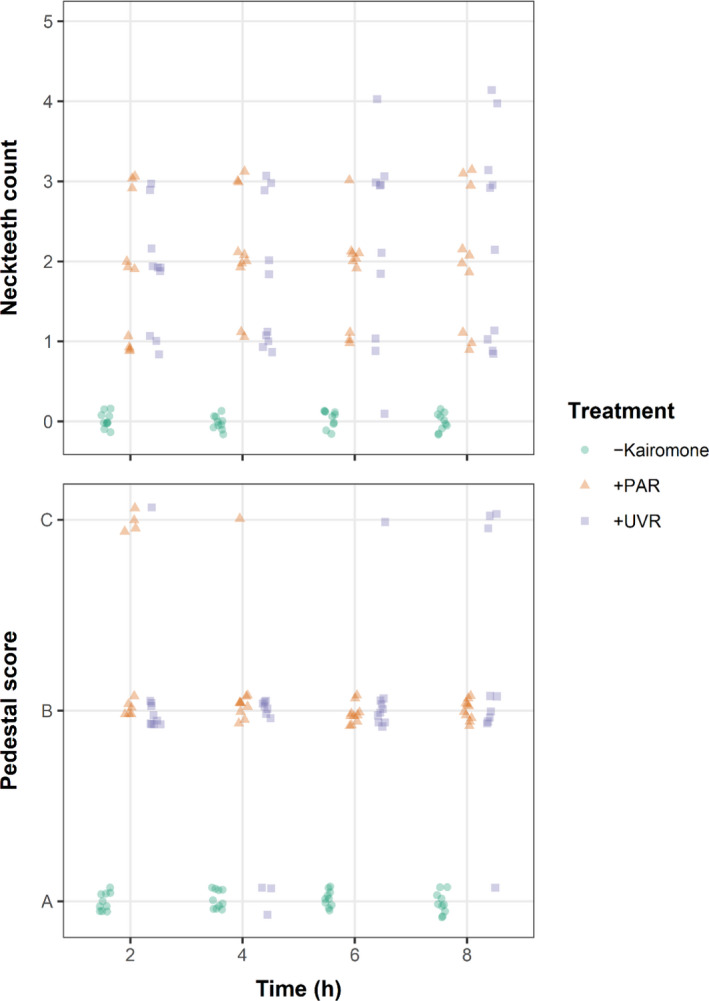
Number of neckteeth and pedestal score in 2nd instar offspring of *Daphnia pulex* mothers of clone UNI. In the UVR and PAR treatment, media‐filled jars with added kairomone extract have been exposed to UVR and PAR, respectively, for 2, 4, 6, and 8 hr before placing *D. pulex* mothers in the jars. In the control treatment “–Kairomone”, *D. pulex* mothers were kept in jars in the dark without addition of kairomone extract. Neckteeth count and pedestal scores of individual offspring juveniles (*n* = 10 per treatment group and time interval) are shown. A: No pedestal, B: small pedestal, C: large pedestal

Our first experiment showed that the prepared *Chaoborus* kairomone extract was effective in inducing neckteeth and pedestals in the 2nd instar of the *D. pulex* UNI clone (Figure 2, Appendix: Figure A1) as there was a significant difference between treatments receiving kairomone (i.e., PAR and UVR treatments) and the control that received no kairomones (Appendix: Table A1). Notably, this experiment also showed no difference in neckteeth count and pedestal score between the UVR and PAR treatment (Figure 2, Appendix: Table A1), indicating that UVR exposure of the kairomone suspensions of up to 8 hr did not have a negative effect on neckteeth and pedestal development.

### 3.2 Effects of kairomones and UVR on size measurements

**FIGURE 3:**
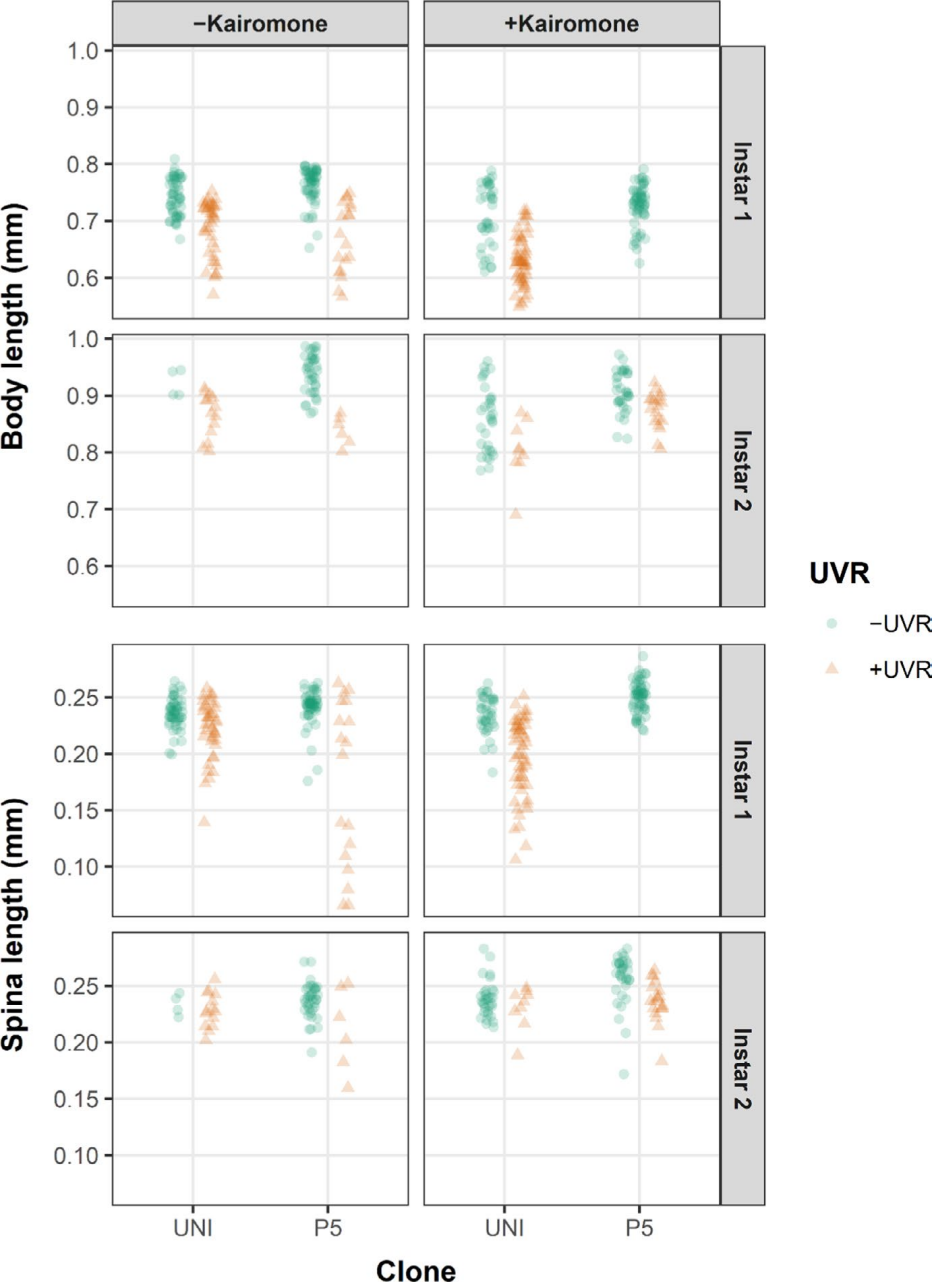
Body length and tail spine (spina) length of two *Daphnia pulex* clones (UNI, P5) in instar 1 and instar 2 juveniles with and without exposure to kairomone, and with exposure to UVR (+UVR) and with exposure to PAR (–UVR). There are no data available for clone P5 instar 1 juveniles in the +kairomone/+UVR treatment

**FIGURE 4:**
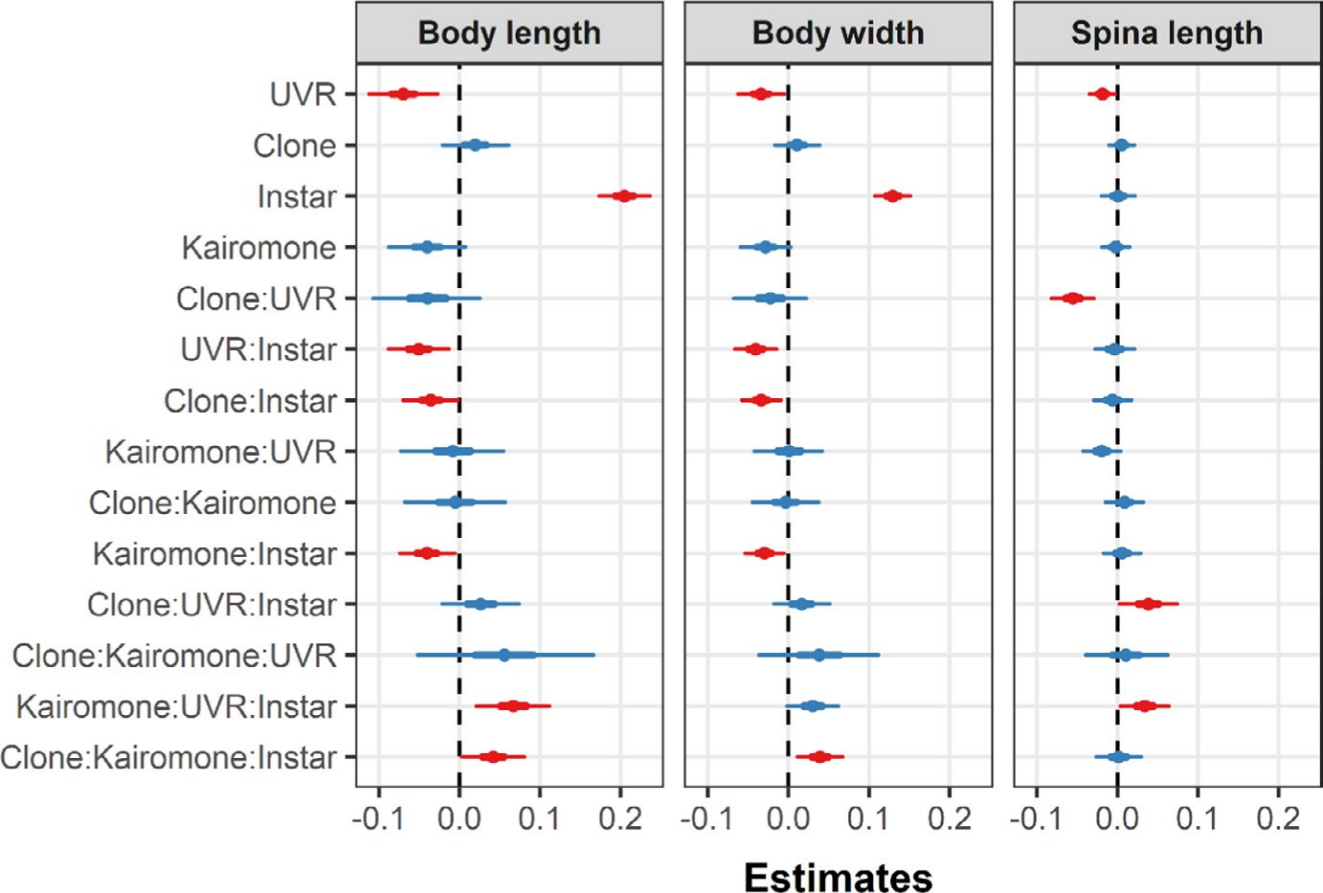
Summary of the predictor (“fixed”) effects for the final (reduced) three‐variate model of body length, body width, and spina length. All available data from both clones and instars were used. The thick and thin lines describe the 50% and 95% credible intervals (CI) of each parameter, respectively. Effects were considered strong (indicated in red) when their 95% CI did not include 0

**FIGURE A2:**
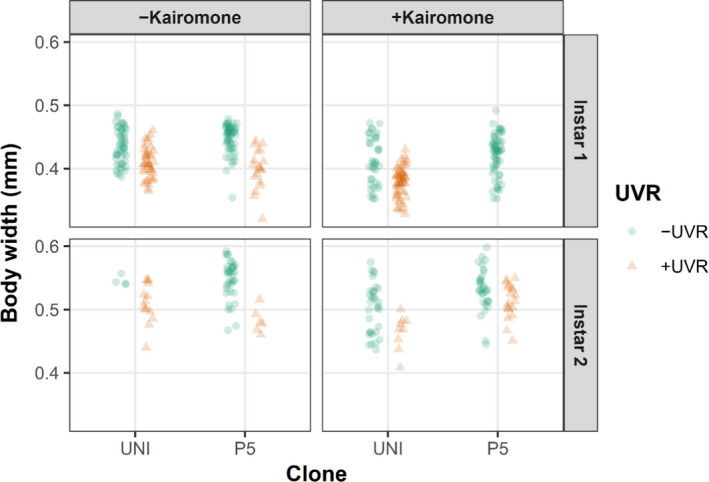
Body width of two *Daphnia pulex* clones (UNI, P5) in instar 1 and instar 2 juveniles with and without exposure to kairomone, and with exposure to UVR (+UVR) and with exposure to PAR (–UVR). There are no data available for clone P5 instar 1 juveniles in the +kairomone treatment

**FIGURE A3:**
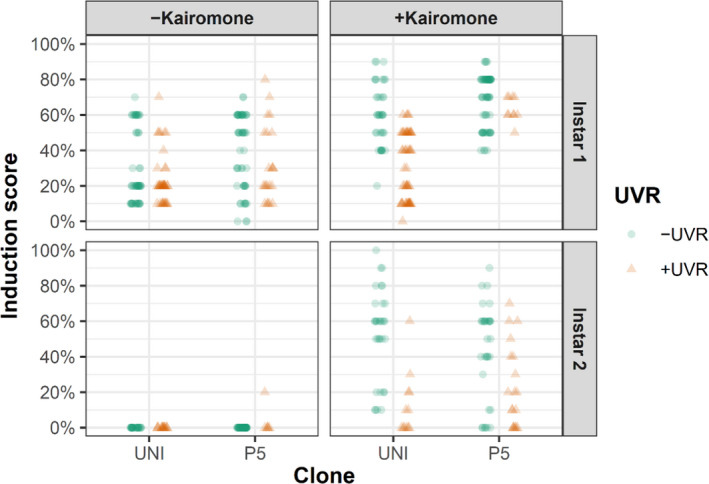
Neckteeth induction score of two *Daphnia pulex* clones (UNI, P5) in instar 1 and instar 2 juveniles with and without exposure to kairomone, and with exposure to UVR (+UVR) and with exposure to PAR (–UVR)

Across all treatments, body length of 1st instar juveniles ranged from 0.55 to 0.8 mm, whereas body length of 2nd instar juveniles was larger and ranged generally from 0.75 to 1.0 mm (Figure 3). Body length in both instars was not affected by the kairomone treatment, but UVR exposure led to decreased body length, especially in the treatment without kairomones (Figures 3 and 4). Body width was highly correlated with body length and was thus affected similarly (Appendix: Figure A2). Tail spine (spina) length was similar between instars and showed high variability in instar 1 (Figures 3 and 4). Spina length in both instars was not affected by the kairomone treatment, but negatively affected by UVR (Figure 4). Spina length of instar 1 juveniles in both kairomone treatments seemed to be lower under UVR exposure than without UVR exposure (Figure 3).

**FIGURE A4:**
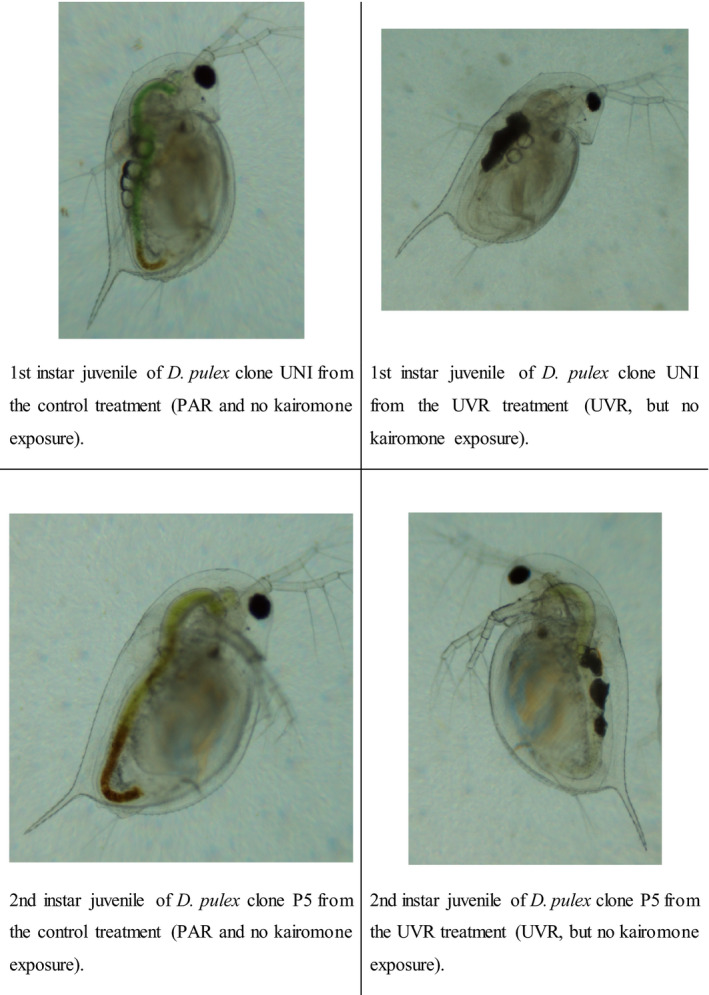
Comparison of ‘healthy’ animals that were exposed to PAR (i.e., no UVR) with animals from the UVR exposure treatment that showed signs of lipid store damage

Microscopic inspection revealed that juveniles not exposed to UVR appeared healthy, contrary to many juveniles in the UVR treatments (Appendix: Figure A4). Furthermore, all juveniles developed normally and survived until instar 2 in the absence of UVR, while many juveniles in the UVR treatments failed to develop from instar 1 to instar 2 or died (mortality of 50% or higher), which limited measurements for instar 2 individuals in the UVR treatments.

### 3.3 Effects of kairomones and UVR on morphological defense traits

**FIGURE 5:**
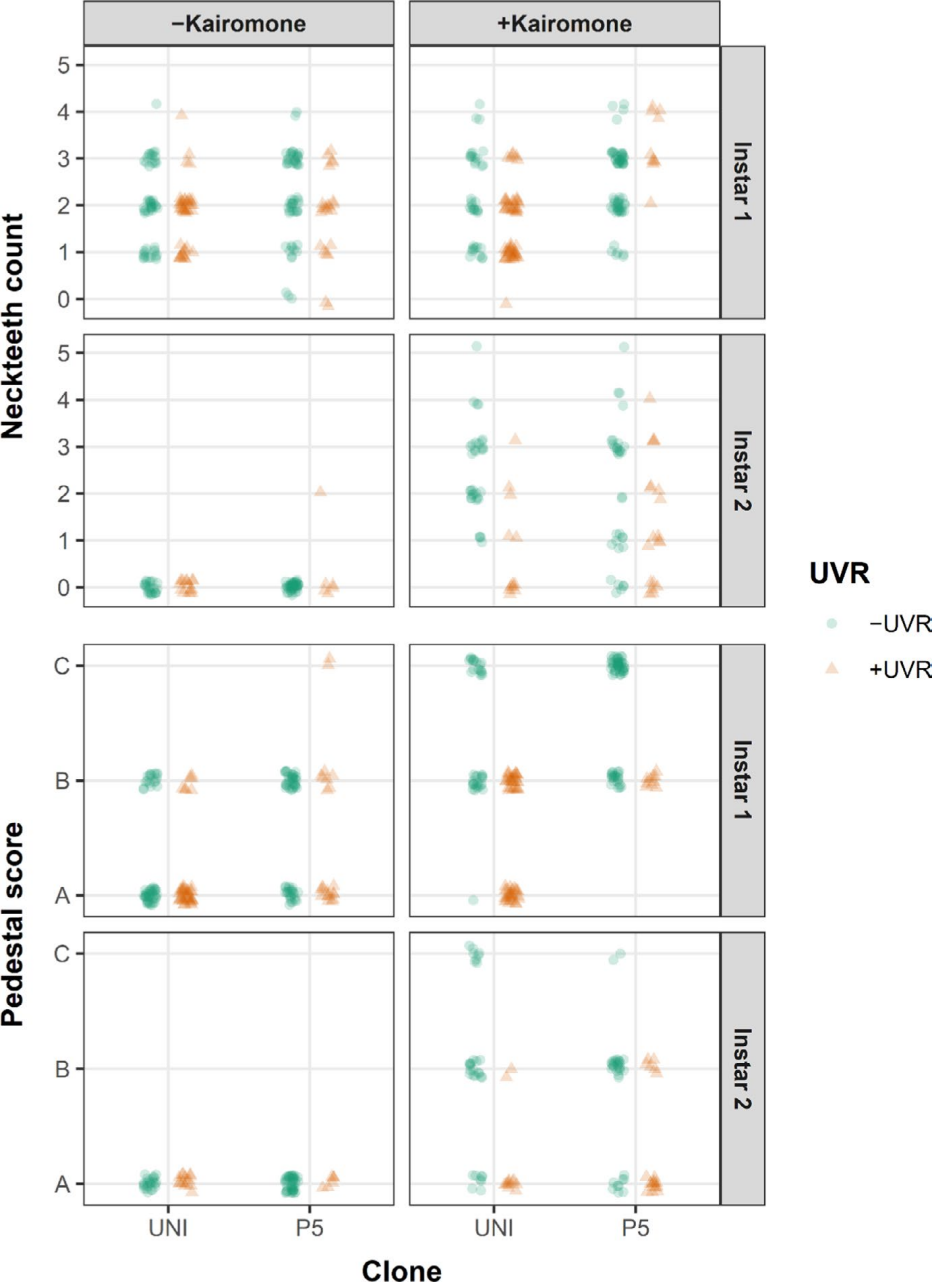
Number of neckteeth and pedestal score of two *Daphnia pulex* clones (UNI, P5) in instar 1 and instar 2 juveniles with and without exposure to kairomone, and with exposure to UVR (+UVR) and with exposure to PAR (–UVR). A: No pedestal, B: small pedestal, C: large pedestal

**FIGURE 6:**
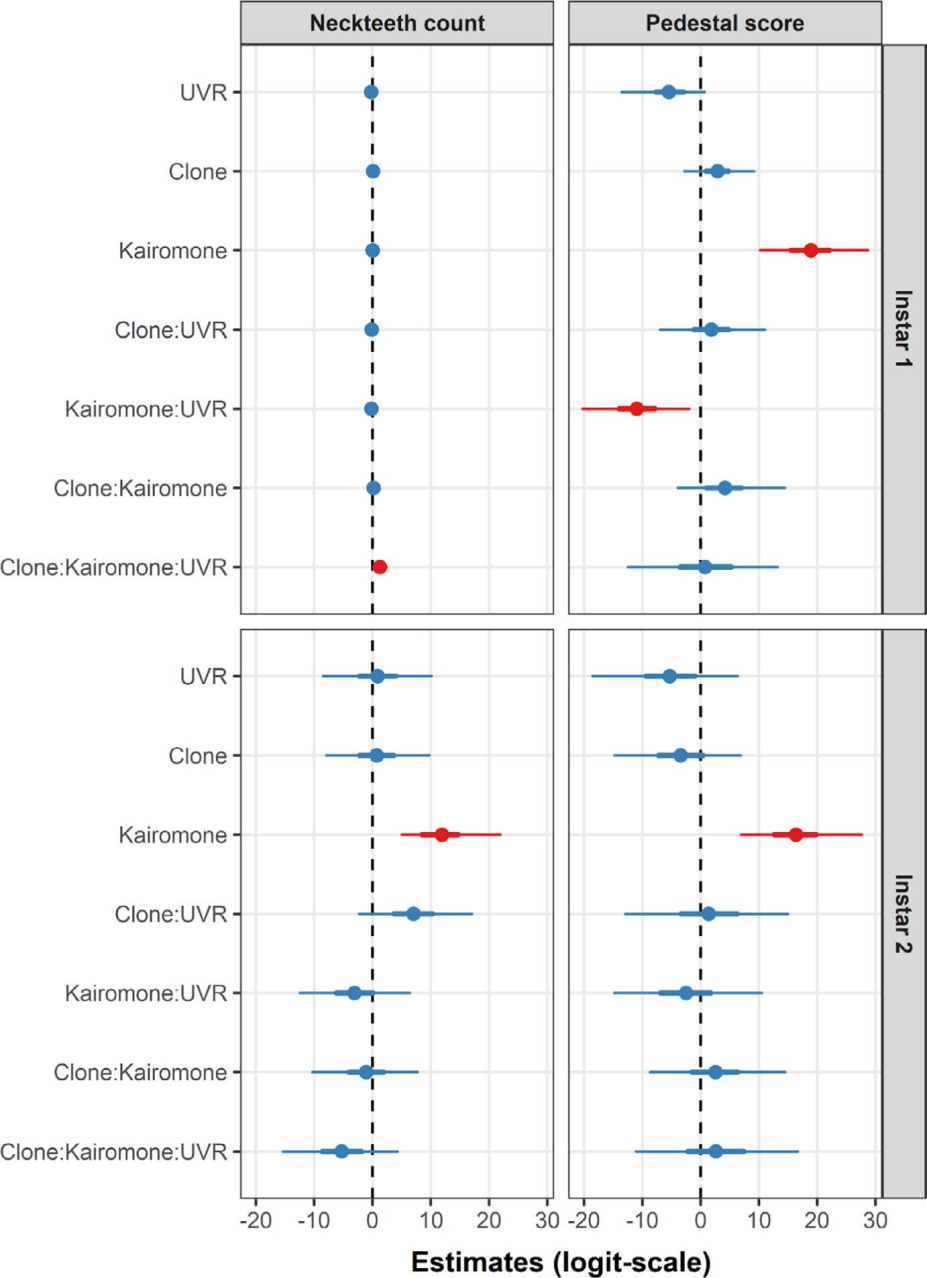
Summary of the predictor (“fixed”) effects for the final (reduced) bivariate model of neckteeth count and pedestal score for instar 1 and instar 2. All available data from both clones and instars were used; however, separate models were fit for each instar. The thick and thin lines describe the 50% and 95% credible intervals (CI) of each parameter, respectively. Effects were considered strong (indicated in red) when their 95% CI did not include 0

Instar 1 juveniles of both clones showed a similar range in the numbers of neckteeth without or with kairomone exposure across UVR treatments (Figure 5), suggesting neckteeth expression in the 1st instar as a constitutive rather than an inducible defense. Instar 1 juveniles showed clone‐specific differences that were dependent on kairomone treatment and UVR exposure (Figure 6). Without kairomones, instar 1 juveniles of both clones developed mostly 1–3 neckteeth irrespective of the UVR treatment (Figure 5). With kairomone exposure, the UNI clone showed a slightly reduced neckteeth number under UVR exposure, whereas the P5 clone show a slightly increased number of neckteeth under UVR exposure (but note the low number of data points for P5 in the UVR treatment with kairomone, Figure 5).

Instar 2 juveniles show the typical pattern of inducible defenses, where neckteeth were observed only under kairomone exposure (Figure 5). Most 2nd instar juveniles of the UNI clone in the kairomone treatment developed no neckteeth under UVR exposure, but 2–3 neckteeth without UVR exposure (Figure 5), suggesting a strongly negative effect of UVR on neckteeth induction. The 2nd instar juveniles of the P5 clone in the kairomone treatment developed mostly 0–2 neckteeth and without UVR exposure mostly 1 or 3 neckteeth (Figure 5), suggesting a weaker negative effect of UVR on neckteeth induction of this clone. The described responses for instar 2 juveniles and the high within treatment variability of neckteeth counts resulted in weak interactive effects of kairomone, clone, and UVR (Figure 6).

Without UVR exposure, instar 1 juveniles of both clones showed a stronger pedestal development under kairomone exposure than without kairomone exposure (Figure 5): with kairomones, only medium and large pedestals (score B and C) were observed, whereas without kairomones, only medium or no pedestals were formed (score A and B). This pattern changed with UVR exposure, where the development of large pedestals disappeared in the kairomone treatment (Figure 5), indicating a strong suppressive effect of UVR on pedestal development for instar 1 individuals (Figure 6). Instar 2 juveniles of both clones showed a similar but weaker suppressive effect of UVR on pedestal development in the kairomone treatment (Figure 6), without UVR exposure a large portion of juveniles developed medium pedestals, while with UVR exposure the majority of juveniles developed no pedestals (Figure 5).

## 4 DISCUSSION

Our results show that UVR, in the intensity, wavelengths, and duration applied, did not denature kairomones to levels that influenced the effectivity in inducing morphological defense traits (see Figure 2). This seemingly contrasts Sterr and Sommaruga (2008), who have shown that strong UVR exposure of *Chaoborus* kairomones can make the kairomones ineffective in inducing defensive traits in *D. pulex*. There are some reasons that could have led to this observed deviation, even though both studies used UV‐A lamps with probably similar wavelength spectra, for example, peak emission at 340 nm. First, the dose and dose rate of UVR applied in our study were likely substantially lower. Sterr and Sommaruga (2008) provided integrated irradiance only between 280 and 320 nm (even though lamps had a maximum emission at 340 nm), making it difficult to compare irradiance doses between this and our study. Their estimated integrated irradiance corresponded to a final dose that was equivalent to a typical daily integrated value for summer at mid latitudes (Sterr & Sommaruga, 2008), whereas our applied irradiance of 1,900 lux for a maximum of 8 hr was lower, corresponding rather to doses obtained at overcast sky in autumn or winter.

Second, the concentration of kairomone extract applied in our study could have been at such a high level that potential denaturation of kairomones did not decrease kairomone concentration below levels of maximum induction. It has been shown that neckteeth induction is dose dependent, with a sharp threshold between concentrations that do or do not induce neckteeth (Dennis et al., 2011; Hammill et al., 2008). The *Chaoborus* kairomone concentration in our study could have been higher than the concentration in Sterr and Sommaruga (2008) as we used a concentrated kairomone extract compared to the previous study in which live *Chaoborus* larvae were incubated to obtain a batch of kairomone‐conditioned medium.

*Chaoborus* kairomone molecules are carbon‐based, water‐soluble molecules composed of long‐chained (>C14) fatty acids (Weiss, Albada, et al., 2018) and thus likely fairly resistant to denaturation. In natural waters, kairomones may however be degraded by bacteria (Beklioglu et al., 2006), but the very short time period required for neckteeth induction of only a few hours (Naraki et al., 2013; Weiss et al., 2016) may limit the impact of degradation. Moreover, in the presence of *Chaoborus*, kairomones will be constantly produced, likely counteracting the degrading effects of bacteria and UVR. It should also be remarked that in most natural waters, even those with modest concentrations of colored dissolved organic matter (cDOM), only the upper wavelength range of UVR close to visible light (as applied in our study) may penetrate to ecologically relevant depths.

The UVR‐regime applied in our study has previously been shown to provide negative impacts on *Daphnia*, primarily due to DNA damage caused by ambient induction of free radicals (Wolf et al., 2017). The results of our study showed that juvenile *D. pulex* responded mostly adversely to UVR both for body size traits (Figure 3) and for induced morphological defense traits under predation threat (Figure 5). While the effects under predation threat on neckteeth number were clone‐specific in instar 1 and weak in instar 2, UVR exposure led to smaller pedestals, especially in instar 1 (Figure 5). This shows that UVR indeed may impact *D. pulex's* capability to form morphological defenses. Moreover, body size traits, that is, body length, body width, and tail spine length, were smaller in UVR‐exposed than in nonexposed animals across all kairomone treatments and instars (except for spina length in the 2nd instar, Figure 3). This is an important additional finding, as smaller animals need longer time to grow out of the “predation size window” of the gape‐limited *Chaoborus* larvae (Riessen et al., 2012; Riessen & Trevett‐Smith, 2009), making UVR‐exposed animals longer susceptible to predation.

Juvenile *D. pulex* are susceptible to predation by the gape‐limited *Chaoborus* larvae only in a certain body size range, because this predator has to “swallow” its prey as a whole (e.g., Swift, 1992). In this susceptible “window” of body size, which depends on *Chaoborus* species and larval instar (Riessen & Trevett‐Smith, 2009; Swift, 1992) juveniles of *D. pulex* react with predator‐induced defenses, that is, neckteeth and associated defenses (Riessen et al., 2012; Tollrian, 1993). The neckteeth and associated morphological defenses are no ultimate protection against predation, but increase the likelihood to escape a predation attack, that is, reducing the strike efficiency of *Chaoborus* larvae (Riessen & Trevett‐Smith, 2009). The effectiveness of neckteeth also depends on the size of the juvenile daphnids as the neckteeth become less effective on smaller juveniles (Riessen & Trevett‐Smith, 2009). *D. pulex* in our collection area may often co‐occur with *Chaoborus crystalinus* or *C. flavicans* larvae and the susceptible body size of juveniles is in the range of 0.55–1.2 mm (Swift, 1992). The observed body size in the 1st and 2nd instar of our *D. pulex* juveniles (0.55–1 mm, Figure 3) falls well within this susceptible size range.

The observed neckteeth development in 1st instar juveniles without kairomone exposure is a phenomenon that has been observed also in other *D. pulex* clones (e.g., Naraki et al., 2013; Weiss et al., 2016). This can be seen as a constitutive defense, as the 1st instar juveniles hatch in the susceptible size range, and may have evolved as an adaptation to high predation risk by *Chaoborus* larvae. This might be the case for our clones that probably originate from meta‐populations distributed across small ponds, which are usually devoid of fish but have a high chance of *Chaoborus* occurrence. The observed invariance of neckteeth number to kairomone exposure also suggests canalization, defined as the robustness of a phenotypic trait to environmental variation (Waddington, 1942).

Juveniles from the UVR treatment were often smaller and showed sometimes very small tail spines in instar 1 compared to juveniles from treatments without UVR exposure. Additionally, most UVR‐treated juveniles also appeared very unhealthy (e.g., pale) and showed dark/black lipid droplets within their body (see Appendix). UVR may have damaged energy reserves (lipid droplets) already in the embryonic development phase, that is, when mothers carrying embryos were exposed to UVR. Damage of lipid stores is not unlikely considering that UVR exposure can lead to lipid peroxidation caused by free radicals and reactive oxygen species (ROS), such as hydrogen peroxide (Rautio & Tartarotti, 2010; Souza et al., 2007). Thus, UVR may have impaired embryo development in the brood pouch, which resulted in smaller hatchlings observed in the UVR treatment. Many planktonic crustaceans induce defenses against such UVR‐related stress by synthesizing or accumulating photoprotective compounds (Bashevkin et al., 2020; Herbert & Emery, 1990; Hylander et al., 2015). However, previous work using another *Daphnia*–predator system has demonstrated that resistance of the melanic *D. dadayana* to UVR is not affected by the investment in antipredatory defenses (Wolinski et al., 2020).

We also observed that many juveniles in the UVR treatments could not develop into instar 2 or died (mortality of 50% or higher), which limited the length measurements and scoring for 2nd instar individuals. Even though the applied UVR is considered low compared with potential surface radiation, it is a realistic dose in the upper water column since UVR is attenuated rapidly with depth. Lethal stress from using small experimental flasks is unlikely, because we did not observe mortality in the current and similar setups without UVR exposure (Sperfeld et al., 2020). A potential explanation for the observed detrimental effect of the applied moderate UVR exposure could be that the animals were not able to escape the UVR stress by moving into deeper water layers as they can do in deeper, natural water bodies.

Decreased induction of defense traits in the UVR treatment could also indicate a shift in energy allocation from antipredatory defenses to cellular defenses against UVR damage. The notion that metabolic costs incur during acclimation to UVR stress may explain organisms’ reduced capacity to deal with other stressors (Kim et al., 2009). As antipredatory defenses are adaptive under predation pressures (Harvell, 1990), potential fitness costs of defense trait formation may have become a limiting factor when exposed to the additional UVR stressor. The allocation of energy to different functions when under threat of multiple stressors may have a significant effect on morphology and life history. Alternatively, changes in environmental stressors, such as elevated levels in freshwater *p*CO_2_, can alter chemical communication between predator and prey by reducing the ability of *D. pulex* to sense the *Chaoborus* kairomone, resulting in a reduction of neckteeth formation (Weiss, Pötter, et al., 2018).

We also found differences between the two tested clones in their responses to UVR and kairomone exposure, though the effects were smaller compared to the effects of either stressor alone. Moreover, expression of inducible defenses for the clone from the shaded, deeper artificial pond (UNI) seemed to be more adversely affected by UVR exposure than for the clone originating from the shallow, light‐exposed rockpool (P5). The capability of the usually stronger light‐exposed rockpool clone to better resist UVR stress may reflect some adaptation to its original environment. However, many more *D. pulex* clones from habitats differing in the intensity of UVR exposure and/or *Chaoborus* predation would be needed to verify how common such interclonal differences are. The *D. pulex* clones used in this experiment were obtained from water bodies with no signs of *Chaoborus* predator occurrence, at least at the time of sampling. The potential lack of *Chaoborus* larvae in the original ponds did not affect the investigated clones’ ability to form neckteeth when induced in the laboratory experiments. The ability of *Daphnia* to adapt to changing environmental conditions by shifts in gene expression has allowed for the distribution of *Daphnia* to inhabit an array of different water bodies. Phenotypic plasticity has provided the necessary tools for *Daphnia* to respond to shifts in predator dynamics, environmental stressors, and food availability (Thiel & Wellborn, 2018).

The most striking morphological response of *D. pulex* juveniles to kairomones of *Chaoborus* larvae consists of two parts: the expression of neckteeth and the development of a pedestal beneath the neckteeth (e.g., Tollrian, 1993). Most studies since Tollrian (1993) have adopted his neckteeth induction score algorithm, which combines the pedestal stage classification and the neckteeth count into a single numerical index ranging from 0% to 100%. Unfortunately, Tollrian's induction score violates the normal distribution assumption behind standard regression and ANOVA. Continuous variables constrained to the unit interval (0 to 1, or equivalently 0%–100%) are more compatible with the beta distribution, which has support only on this interval, than the normal, which is defined over the entire real line (Kieschnick & McCullough, 2003). Moreover, many studies report zero‐inflated score distributions with an overabundance of no‐induction zeros, which are even less compatible with the normal distribution. In the present study, we use an alternate approach where we fit a bivariate Bayesian model directly to the observed pedestal classes and neckteeth counts. The main advantage is that we can make direct predictions of pedestal class probabilities and expected neckteeth counts, without introducing arbitrary weighting between the two, as in Tollrian's algorithm, and that we can express contrasts and effect sizes on a log odds ratio scale. A possible disadvantage is that we introduce a more complex computational procedure for the analysis, although this is to some extent alleviated by the development of powerful Bayesian computational engines like *Stan* (Carpenter et al., 2017) and user‐friendly front‐end packages like *brms* (Bürkner, 2017). We use the same Bayesian approach to fit a multivariate normal model for all morphometric responses (body length, body width, and spina length; Figure 4), as an alternative to fitting separate models for each of these attributes.

Levels of UVR are still elevated compared to the mid‐1950s, after the Montreal Protocol 1987 initiated stopping emissions of chlorofluorocarbon compounds that eroded the ozone layer at that time (Dugo et al., 2012; Williamson et al., 2014). Even after recovery of the ozone layer, UVR is not necessarily predicted to decrease in the future (Bais et al., 2018; Williamson et al., 2014). In the most populated areas of the northern hemisphere, UVR is predicted to increase due to expected improvement of air quality and reductions of aerosols (Bais et al., 2015). There is also the possibility of an increase in aquatic environments due to climate warming related decreases in ice cover (Williamson et al., 2014), though there is also the possibility of reduced UVR in waters undergoing browning due to attenuation of radiation by cDOM (Williamson et al., 1996).

## 5 CONCLUSION

Elevated UVR leads to detrimental effects on key taxa in aquatic systems (Llabrés et al., 2013; Peng et al., 2017). *D. pulex* found in clear and shallow lakes and ponds are subject to significant UVR stress, which could affect both the fitness and survival of this key species that is both an important grazer on phytoplankton and an important forage prey for many invertebrate predators and fish (Miner Brooks et al., 2012). Our study aimed to address the possibility as to whether or not UVR had a synergistic or antagonistic effect on predator cue‐induced defense traits. The results showed that UVR had mostly an antagonistic effect on the induction of morphological defense traits under predation threat and that this effect was stronger in the 1st than in the 2nd instar. UVR exposure of *D. pulex* under predation threat led to clone‐dependent effects on neckteeth number in instar 1, but clearly to smaller pedestals in juveniles across both instars. Moreover, UVR exposure decreased body length and width in both instars, and also often spina length across both kairomone treatments. The net effect of the combination of mostly reduced morphological defense traits and smaller body size traits makes UVR‐exposed juveniles very likely more susceptible to *Chaoborus* predation. Modeling studies have shown that inducible defenses are among the ecological factors that promote stability in multitrophic communities (Verschoor et al., 2004; Vos et al., 2004). Thus, the inability of *D. pulex* to develop induced morphological defenses against predators and a reduced body size under elevated UVR could have serious implications on community composition, food web structure, and ultimately the entire ecosystem of which *D. pulex* is part of.

## CONFLICT OF INTEREST

None declared.

## AUTHOR CONTRIBUTION

**Franceen Eshun‐Wilson:** Conceptualization (lead); Data curation (lead); Formal analysis (equal); Investigation (equal); Methodology (equal); Resources (equal); Software (equal); Validation (equal); Visualization (equal); Writing‐original draft (equal); Writing‐review & editing (equal). **Raoul Wolf:** Conceptualization (equal); Data curation (equal); Formal analysis (lead); Investigation (equal); Methodology (equal); Resources (equal); Software (equal); Supervision (equal); Validation (equal); Visualization (equal); Writing‐review & editing (equal). **Tom Andersen:** Conceptualization (equal); Data curation (equal); Formal analysis (equal); Investigation (equal); Methodology (equal); Project administration (equal); Resources (equal); Software (equal); Supervision (equal); Validation (lead); Visualization (equal); Writing‐original draft (equal); Writing‐review & editing (equal). **Dag O. Hessen:** Conceptualization (equal); Funding acquisition (lead); Investigation (equal); Methodology (equal); Project administration (equal); Resources (equal); Supervision (lead); Validation (equal); Visualization (equal); Writing‐original draft (equal); Writing‐review & editing (equal). **Erik Sperfeld:** Conceptualization (equal); Data curation (equal); Formal analysis (equal); Funding acquisition (equal); Investigation (equal); Methodology (equal); Project administration (equal); Supervision (equal); Validation (equal); Visualization (equal); Writing‐original draft (lead); Writing‐review & editing (equal).

## Data Availability

All data and scripts have been made publicly available on Dryad (https://doi.org/10.5061/dryad.kprr4xh3c) and additionally on GitHub (https://github.com/RaoulWolf/UV‐radiation‐affects‐anti‐predatory‐defense‐traits‐in‐Daphnia‐pulex).

## Appendix 1

**TABLE A1:** Results of statistical tests for the ‘experiment testing the effect of UVR on kairomone effectivity’. Differences were considered significant for posterior probabilities below .05. The credible interval (CI) of the estimates is given for 95%

Endpoint	Test	Estimate	Standard error	Lower CI	Upper CI	Posterior probability
Pedestal score	PAR – UVR = 0	0.66	0.63	−0.52	1.93	>.05
	PAR − Control = 0	10.81	3.11	4.84	26.4	<.001***
	UVR – Control = 0	10.16	3.10	4.40	25.1	<.001***
Neck teeth count	PAR − UVR = 0	−0.08	0.20	−0.48	0.32	>.05
	PAR − Control = 0	9.11	3.45	3.35	28.5	<.001***
	UVR – Control = 0	9.20	3.45	3.47	28.8	<.001***

## References

[ece36999-bib-0001] Abrams, P. A. (1986). Adaptive responses of predators to prey and prey to predators: The failure of the arms‐race analogy. Evolution, 40(6), 1229–1247. 10.1111/j.1558-5646.1986.tb05747.x 28563514

[ece36999-bib-0002] Bais, A. F. , Lucas, R. M. , Bornman, J. F. , Williamson, C. E. , Sulzberger, B. , Austin, A. T. , Wilson, S. R. , Andrady, A. L. , Bernhard, G. , McKenzie, R. L. , Aucamp, P. J. , Madronich, S. , Neale, R. E. , Yazar, S. , Young, A. R. , de Gruijl, F. R. , Norval, M. , Takizawa, Y. , Barnes, P. W. , … Heikkilä, A. M. (2018). Environmental effects of ozone depletion, UV radiation and interactions with climate change: UNEP Environmental Effects Assessment Panel, update 2017. Photochemical & Photobiological Sciences, 17(2), 127–179. 10.1039/c7pp90043k 29404558 PMC6155474

[ece36999-bib-0003] Bais, A. F. , McKenzie, R. L. , Bernhard, G. , Aucamp, P. J. , Ilyas, M. , Madronich, S. , & Tourpali, K. (2015). Ozone depletion and climate change: Impacts on UV radiation. Photochemical & Photobiological Sciences, 14, 19–52. 10.1039/C4PP90032D 25380284

[ece36999-bib-0004] Bashevkin, S. M. , Christy, J. H. , & Morgan, S. G. (2020). Adaptive specialization and constraint in morphological defences of planktonic larvae. Functional Ecology, 34, 217–228. 10.1111/1365-2435.13464

[ece36999-bib-0005] Beklioglu, M. , Cetin, A. G. , Zorlu, P. , & Ay‐Zog, D. (2006). Role of planktonic bacteria in biodegradation of fish‐exuded kairomone in laboratory bioassays of diel vertical migration. Archiv Für Hydrobiologie, 165, 89–104. 10.1127/0003-9136/2006/0165-0089

[ece36999-bib-0006] Boeing, W. J. , Ramcharan, C. W. , & Riessen, H. P. (2006). Clonal variation in depth distribution of *Daphnia pulex* in response to predator kairomones. Archiv Für Hydrobiologie, 166(2), 241–260. 10.1127/0003-9136/2006/0166-0241

[ece36999-bib-0007] Bürkner, P.‐C. (2017). brms: An R Package for Bayesian Multilevel Models Using Stan. Journal of Statistical Software, 80(1), 1–28. 10.18637/jss.v080.i01

[ece36999-bib-0008] Carpenter, B. , Gelman, A. , Hoffman, M. D. , Lee, D. , Goodrich, B. , Betancourt, M. , Brubaker, M. , Guo, J. , Li, P. , & Riddell, A. (2017). Stan: A probabilistic programming language. Journal of Statistical Software, 76(1), 1–32. 10.18637/jss.v076.i01 PMC9788645 36568334

[ece36999-bib-0063] Carter, M. J. , Lind, M. I. , Dennis, S. R. , Hentley, W. , & Beckerman, A. P. (2017). Evolution of a predator‐induced, nonlinear reaction norm. Proceedings of the Royal Society B: Biological Sciences, 284(1861). 10.1098/rspb.2017.0859 PMC5577476 28835554

[ece36999-bib-0009] Christjani, M. , Fink, P. , & von Elert, E. (2016). Phenotypic plasticity in three *Daphnia* genotypes in response to predator kairomone: Evidence for an involvement of chitin deacetylases. Journal of Experimental Biology, 219(11), 1697–1704. 10.1242/jeb.133504 26994174

[ece36999-bib-0010] Clay, K. , & Kover, P. X. (1996). The red queen hypothesis and plant pathogen interactions. Annual Review of Phytopathology, 34(1), 29–50. 10.1146/annurev.phyto.34.1.29 15012533

[ece36999-bib-0011] Cullen, J. J. , & Neale, P. J. (1994). Ultraviolet radiation, ozone depletion, and marine photosynthesis. Photosynthesis Research, 39(3), 303–320. 10.1007/BF00014589 24311127

[ece36999-bib-0012] Dennis, S. R. , Carter, M. J. , Hentley, W. T. , & Beckerman, A. P. (2011). Phenotypic convergence along a gradient of predation risk. Proceedings of the Royal Society B: Biological Sciences, 278(1712), 1687–1696. 10.1098/rspb.2010.1989 PMC3081771 21084350

[ece36999-bib-0013] Dickey, J. M. (1971). The weighted likelihood ratio, linear hypotheses on normal location parameters. The Annals of Mathematical Statistics, 42(1), 204–223. 10.1214/aoms/1177693507

[ece36999-bib-0014] Dodson, S. I. (1989). The ecological role of chemical stimuli for the zooplankton: Predator‐induced morphology in *Daphnia* . Oecologia, 78(3), 361–367. 10.1007/BF00379110 28312582

[ece36999-bib-0015] Dugo, M. A. , Han, F. , & Tchounwou, P. B. (2012). Persistent polar depletion of stratospheric ozone and emergent mechanisms of ultraviolet radiation‐mediated health dysregulation. Reviews on Environmental Health, 27(2–3), 103–116. 10.1515/reveh-2012-0026 23023879 PMC3768272

[ece36999-bib-0016] Ghalambor, C. K. , Hoke, K. L. , Ruell, E. W. , Fischer, E. K. , Reznick, D. N. , & Hughes, K. A. (2015). Non‐adaptive plasticity potentiates rapid adaptive evolution of gene expression in nature. Nature, 525(7569), 372–375. 10.1038/nature15256 26331546

[ece36999-bib-0061] Guillard, R. R. (1975). Cultures of phytoplankton for feeding of marine invertebrates. W. L. Smith & M. H. Chanley (Eds.), Culture of marine invertebrate animals (pp. 26–60). Plenum Press.

[ece36999-bib-0017] Hammill, E. , Rogers, A. , & Beckerman, A. P. (2008). Costs, benefits and the evolution of inducible defences: A case study with *Daphnia pulex* . Journal of Evolutionary Biology, 21(3), 705–715. 10.1111/j.1420-9101.2008.01520.x 18355186

[ece36999-bib-0018] Hansson, L.‐A. , Hylander, S. , & Sommaruga, R. (2007). Escape from UV threats in zooplankton: A cocktail of behavior and protective pigmentation. Ecology, 88(8), 1932–1939. 10.1890/06-2038.1 17824423

[ece36999-bib-0019] Harvell, C. D. (1990). The ecology and evolution of inducible defenses. The Quarterly Review of Biology, 65(3), 323–340. 10.1086/416841 2236483

[ece36999-bib-0020] Hebert, P. D. N. , & Grewe, P. M. (1985). Induced shifts in the morphology of *Daphnia ambigua* . Limnology and Oceanography, 30, 1291–1297.

[ece36999-bib-0021] Herbert, P. D. N. , & Emery, C. J. (1990). The adaptive significance of cuticular pigmentation in *Daphnia* . Functional Ecology, 4, 703–710. 10.2307/2389739

[ece36999-bib-0022] Hessen, D. O. , & Rukke, N. A. (2000). UV radiation and low calcium as mutual stressors for *Daphnia* . Limnology and Oceanography, 45, 1834–1838.

[ece36999-bib-0023] Hessen, D. O. , Van Donk, E. , & Andersen, T. (1995). Growth responses, P‐uptake and loss of flagellae in *Chlamydomonas reinhardtii* exposed to UV‐B. Journal of Plankton Research, 17(1), 17–27. 10.1093/plankt/17.1.17

[ece36999-bib-0024] Hoverman, J. T. , & Relyea, R. A. (2009). Survival trade‐offs associated with inducible defences in snails: The roles of multiple predators and developmental plasticity. Functional Ecology, 23(6), 1179–1188. 10.1111/j.1365-2435.2009.01586.x

[ece36999-bib-0025] Hylander, S. , Kiørboe, T. , Snoeijs, P. , Sommaruga, R. , & Nielsen, T. G. (2015). Concentrations of sunscreens and antioxidant pigments in Arctic *Calanus* spp. in relation to ice cover, ultraviolet radiation, and the phytoplankton spring bloom. Limnology and Oceanography, 60, 2197–2206.

[ece36999-bib-0026] Kieschnick, R. , & McCullough, B. D. (2003). Regression analysis of variates observed on (0, 1): Percentages, proportions and fractions. Statistical Modelling, 3(3), 193–213. 10.1191/1471082X03st053oa

[ece36999-bib-0027] Kim, J. , Lee, M. , Oh, S. , Ku, J.‐L. , Kim, K.‐H. , & Choi, K. (2009). Acclimation to ultraviolet irradiation affects UV‐B sensitivity of *Daphnia magna* to several environmental toxicants. Chemosphere, 77(11), 1600–1608. 10.1016/j.chemosphere.2009.09.035 19836821

[ece36999-bib-0066] Klüttgen, B. , Dülmer, U. , Engels, M. , & Ratte, H. T. (1994). ADaM, an artificial freshwater for the culture of zooplankton. Water Research, 28(3), 743–746. 10.1016/0043-1354(94)90157-0

[ece36999-bib-0028] Krueger, D. A. , & Dodson, S. I. (1981). Embryological induction and predation ecology in *Daphnia pulex* . Limnology and Oceanography, 26(2), 219–223. 10.4319/lo.1981.26.2.0219

[ece36999-bib-0029] Lass, S. , & Spaak, P. (2003). Chemically induced anti‐predator defences in plankton: A review. Hydrobiologia, 491, 221–239. 10.1023/A:1024487804497

[ece36999-bib-0030] Llabrés, M. , Agustí, S. , Fernández, M. , Canepa, A. , Maurin, F. , Vidal, F. , & Duarte, C. M. (2013). Impact of elevated UVB radiation on marine biota: A meta‐analysis. Global Ecology and Biogeography, 22, 131–144. 10.1111/j.1466-8238.2012.00784.x

[ece36999-bib-0031] Marrow, P. , Law, R. , & Cannings, C. (1992). The coevolution of predator—prey interactions: ESSS and red queen dynamics. Proceedings: Biological Sciences, 250(1328), 133–141.

[ece36999-bib-0032] Miner Brooks, E. , De Meester, L. , Pfrender, M. E. , Lampert, W. , & Hairston, N. G. (2012). Linking genes to communities and ecosystems: *Daphnia* as an ecogenomic model. Proceedings of the Royal Society B, 279, 1873–1882. 10.1098/rspb.2011.2404 22298849 PMC3311900

[ece36999-bib-0033] Naraki, Y. , Hiruta, C. , & Tochinai, S. (2013). Identification of the precise kairomone‐sensitive period and histological characterization of necktooth formation in predator‐induced polyphenism in *Daphnia pule*x. Zoological Science, 30(8), 619–625. 10.2108/zsj.30.619 23915154

[ece36999-bib-0034] Peng, S. , Liao, H. , Zhou, T. , & Peng, S. (2017). Effects of UVB radiation on freshwater biota: A meta‐analysis. Global Ecology and Biogeography, 26(4), 500–510. 10.1111/geb.12552

[ece36999-bib-0062] R Core Team (2017). R: A language and environment for statistical computing. Available online at: https://www.R‐project.org/

[ece36999-bib-0035] Rautio, M. , & Tartarotti, B. (2010). UV radiation and freshwater zooplankton: Damage, protection and recovery. Freshwater Reviews, 3(2), 105–131. 10.1608/FRJ-3.2.157 21516254 PMC3079903

[ece36999-bib-0036] Riessen, H. P. (2012). Costs of predator‐induced morphological defences in *Daphnia* . Freshwater Biology, 57(7), 1422–1433. 10.1111/j.1365-2427.2012.02805.s

[ece36999-bib-0037] Riessen, H. P. , Linley, R. D. , Altshuler, I. , Rabus, M. , Söllradl, T. , Clausen‐Schaumann, H. , Laforsch, C. , & Yan, N. D. (2012). Changes in water chemistry can disable plankton prey defenses. Proceedings of the National Academy of Sciences of the United States of America, 109, 15377–15382. 10.1073/pnas.1209938109 22949653 PMC3458369

[ece36999-bib-0038] Riessen, H. P. , & Trevett‐Smith, J. B. (2009). Turning inducible defenses on and off: Adaptive responses of *Daphnia* to a gape‐limited predator. Ecology, 90, 3455–3469. 20120813 10.1890/08-1652.1

[ece36999-bib-0039] Sell, A. F. (2000). Morphological defenses induced in situ by the invertebrate predator *Chaoborus*: Comparison of responses between *Daphnia pulex and D. rosea* . Oecologia, 125(1), 150–160. 10.1007/PL00008886 28308217

[ece36999-bib-0040] Souza, M. S. , Modenutti, B. E. , & Balseiro, E. G. (2007). Antioxidant defences in planktonic crustaceans exposed to different underwater light irradiances in Andean lakes. Water, Air, and Soil Pollution, 183, 49–57. 10.1007/s11270-007-9354-8

[ece36999-bib-0041] Sperfeld, E. , Nilssen, J. P. , Rinehart, S. , Schwenk, K. , & Hessen, D. O. (2020). Ecology of predator‐induced morphological defense traits in *Daphnia longispina* (Cladocera, Arthropoda). Oecologia, 192, 687–698. 10.1007/s00442-019-04588-6 31950263 PMC7058565

[ece36999-bib-0042] Sterr, B. , & Sommaruga, R. (2008). Does ultraviolet radiation alter kairomones? An experimental test with *Chaoborus obscuripes* and *Daphnia pulex* . Journal of Plankton Research, 30(12), 1343–1350. 10.1093/plankt/fbn087

[ece36999-bib-0065] Swift, M. C. (1992). Prey capture by the four larval instars of *Chaoborus crystallinus* . Limnology and Oceanography, 37(1), 14–24. 10.4319/lo.1992.37.1.0014

[ece36999-bib-0064] Thiel, M. , & Wellborn, G. A. (2018). The natural history of the crustacea: life histories, Vol. 5. Oxford University Press.

[ece36999-bib-0043] Tollrian, R. (1993). Neckteeth formation in *Daphnia pulex* as an example of continuous phenotypic plasticity: Morphological effects of *Chaoborus* kairomone concentration and their quantification. Journal of Plankton Research, 15(11), 1309–1318. 10.1093/plankt/15.11.1309

[ece36999-bib-0044] Tollrian, R. (1995). Predator‐induced morphological defenses: Costs, life history shifts, and maternal effects in *Daphnia pulex* . Ecology, 76(6), 1691–1705. 10.2307/1940703

[ece36999-bib-0045] Tollrian, R. , & Dodson, S. I. (1999). Inducible defenses in cladocera: Constraints, costs, and multipredator environments. In R. Tollrian , & C. D. Harvell (Eds.), Ecology and evolution of inducible defenses (pp. 177–202). Princeton University Press.

[ece36999-bib-0046] Tollrian, R. , & Harvell, C. D. (1999). The ecology and evolution of inducible defenses. Princeton University Press.

[ece36999-bib-0047] Tollrian, R. , & von Elert, E. (1994). Enrichment and purification of *Chaoborus* kairomone from water: Further steps toward its chemical characterization. Limnology and Oceanography, 39, 788–796.

[ece36999-bib-0048] Vehtari, A. , Gelman, A. , Simpson, D. , Carpenter, B. , & Bürkner, P.‐C. (2019). Rank‐normalization, folding, and localization: An improved R for assessing convergence of MCMC. arXiv:1903.08008v2. https://arxiv.org/abs/1903.08008v2

[ece36999-bib-0049] Verdinelli, I. , & Wasserman, L. (1995). Computing Bayes Factors using a generalization of the Savage‐Dickey Density Ratio. Journal of American Statistical Association, 90, 614–618. 10.1080/01621459.1995.10476554

[ece36999-bib-0050] Verschoor, A. M. , Vos, M. , & Stap, I. V. D. (2004). Inducible defences prevent strong population fluctuations in bi‐ and tritrophic food chains. Ecology Letters, 7(12), 1143–1148. 10.1111/j.1461-0248.2004.00675.x

[ece36999-bib-0051] Vos, M. , Kooi, B. W. , DeAngelis, D. L. , & Mooij, W. M. (2004). Inducible defences and the paradox of enrichment. Oikos, 105, 471–480. 10.1111/j.0030-1299.2004.12930.x

[ece36999-bib-0052] Waddington, C. H. (1942). Canalization of development and the inheritance of acquired characters. Nature, 150, 563–565. 10.1038/150563a0 13666847

[ece36999-bib-0053] Weiss, L. C. , Albada, B. , Becker, S. M. , Meckelmann, S. W. , Klein, J. , Meyer, M. , Schmitz, O. J. , Sommer, U. , Leo, M. , Zagermann, J. , Metzler‐Nolte, N. , & Tollrian, R. (2018). Identification of *Chaoborus* kairomone chemicals that induce defences in *Daphnia* . Nature Chemical Biology, 14(12), 1133–1139. 10.1038/s41589-018-0164-7 30429602

[ece36999-bib-0054] Weiss, L. C. , Heilgenberg, E. , Deussen, L. , Becker, S. M. , Kruppert, S. , & Tollrian, R. (2016). Onset of kairomone sensitivity and the development of inducible morphological defenses in *Daphnia pulex* . Hydrobiologia, 779, 135–145. 10.1007/s10750-016-2809-4

[ece36999-bib-0055] Weiss, L. C. , Pötter, L. , Steiger, A. , Kruppert, S. , Frost, U. , & Tollrian, R. (2018). Rising pCO2 in freshwater ecosystems has the potential to negatively affect predator‐induced defenses in *Daphnia* . Current Biology, 28(2), 327–332.e3. 10.1016/j.cub.2017.12.022 29337079

[ece36999-bib-0056] Williamson, C. E. , Stemberger, R. S. , Morris, D. P. , Frost, T. M. , & Paulsen, S. G. (1996). Ultraviolet radiation in North American lakes: Attenuation estimates from DOC measurements and implications for plankton communities. Limnology and Oceanography, 41, 1024–1034. 10.4319/lo.1996.41.5.1024

[ece36999-bib-0057] Williamson, C. E. , Zepp, R. G. , Lucas, R. M. , Madronich, S. , Austin, A. T. , Ballaré, C. L. , Norval, M. , Sulzberger, B. , Bais, A. F. , McKenzie, R. L. , Robinson, S. A. , Häder, D.‐P. , Paul, N. D. , & Bornman, J. F. (2014). Solar ultraviolet radiation in a changing climate. Nature Climate Change, 4(6), 434–441. 10.1038/nclimate2225

[ece36999-bib-0058] Wolf, R. , Andersen, T. , Hessen, D. O. , & Hylland, K. (2017). The influence of dissolved organic carbon and ultraviolet radiation on the genomic integrity of *Daphnia magna* . Functional Ecology, 31(4), 848–855. 10.1111/1365-2435.12730

[ece36999-bib-0059] Wolf, R. , & Heuschele, J. (2018). Water browning influences the behavioral effects of ultraviolet radiation on zooplankton. Frontiers in Ecology and Evolution, 6, 26. 10.3389/fevo.2018.00026

[ece36999-bib-0060] Wolinski, L. , Modenutti, B. , & Balseiro, E. (2020). Melanin and antipredatory defenses in *Daphnia dadayana* under UVR exposure. International Review of Hydrobiology, 105, 106–114. 10.1002/iroh.201902033

